# Malat1 as an evolutionarily conserved lncRNA, plays a positive role in regulating proliferation and maintaining undifferentiated status of early-stage hematopoietic cells

**DOI:** 10.1186/s12864-015-1881-x

**Published:** 2015-09-03

**Authors:** Xian-Yong Ma, Jian-Hui Wang, Jing-Lan Wang, Charles X Ma, Xiao-Chun Wang, Feng-Song Liu

**Affiliations:** Department of Pathology, Yale University School of Medicine, New Haven, USA; University of Connecticut School of Medicine, Farmington, USA; Department of Surgical Oncology, Affiliated Hospital of Hebei University, Baoding, China; College of Life Sciences, Hebei University, Baoding, China

**Keywords:** Malat1, lncRNA, ATRA, Hematopoiesis, EML, p53

## Abstract

**Background:**

The metastasis-associated lung adenocarcinoma transcription 1 (Malat1) is a highly conserved long non-coding RNA (lncRNA) gene. Previous studies showed that Malat1 is abundantly expressed in many tissues and involves in promoting tumor growth and metastasis by modulating gene expression and target protein activities. However, little is known about the biological function and regulation mechanism of Malat1 in normal cell proliferation.

**Results:**

In this study we conformed that Malat1 is highly conserved across vast evolutionary distances amongst 20 species of mammals in terms of sequence, and found that mouse Malat1 expresses in tissues of liver, kidney, lung, heart, testis, spleen and brain, but not in skeletal muscle. After treating erythroid myeloid lymphoid (EML) cells with All-trans Retinoic Acid (ATRA), we investigated the expression and regulation of Malat1 during hematopoietic differentiation, the results showed that ATRA significantly down regulates Malat1 expression during the differentiation of EML cells. Mouse LRH (Lin-Rhodamine^low^ Hoechst^low^) cells that represent the early-stage progenitor cells show a high level of Malat1 expression, while LRB (Lin **−** Hoechst^Low^ Rhodamine^Bright^) cells that represent the late-stage progenitor cells had no detectable expression of Malat1.

Knockdown experiment showed that depletion of Malat1 inhibits the EML cell proliferation. Along with the down regulation of Malat1, the tumor suppressor gene p53 was up regulated during the differentiation. Interestingly, we found two p53 binding motifs with help of bioinformatic tools, and the following chromatin immunoprecipitation (ChIP) test conformed that p53 acts as a transcription repressor that binds to Malat1’s promoter. Furthermore, we testified that p53 over expression in EML cells causes down regulation of Malat1.

**Conclusions:**

In summary, this study indicates Malat1 plays a critical role in maintaining the proliferation potential of early-stage hematopoietic cells. In addition to its biological function, the study also uncovers the regulation pattern of Malat1 expression mediated by p53 in hematopoietic differentiation. Our research shed a light on exploring the Malat1 biological role including therapeutic significance to inhibit the proliferation potential of malignant cells.

## Background

The long noncoding RNA Malat1 (Metastasis-Associated Lung Adenocarcinoma Transcript 1), also known as NEAT2 (Nuclear-Enriched Abundant Transcript 2) or alpha, is a highly conserved and large-size nuclear noncoding RNA (lncRNA) molecule. This gene was firstly identified in 2003 from early-stage non-small cell lung cancer (NSCLC) cells with highly expression level [1], subsequently a number of publications reported that Malat1 is also as one of the major genes that highly up regulates in different cancers including endometrial cancer [2], breast cancer [3]; cervical cancer [4]; colorectal cancer [5]; hepatocellular carcinoma [6]; liver cancer [7]; neuroblastoma [8]; osteosarcoma [9], pancreatic cancer [10], prostate cancer [11], bladder cancer [12], and gastric cancer [13]. Targeted deletion of Malat1 in human lung tumor cells impairs tumor metastasis in a mouse xenograft model [14]. Therefore, expression of Malat1 correlates with tumor development, progression, metastasis and survival in different cancer types. In addition to the important role in proliferation and metastases of cancers, Malat1 also involves in other diseases and even in normal physiological processes of cells, for example: It has been reported that knockdown of Malat1 using siRNA suppresses myoblast proliferation by arresting cell growth in the G(0)/G(1) phase [15], thus target therapy of this gene shows a potential value for recovery of muscle atrophy or muscle wasting diseases. In addition, Lin M.Y., et al. [16] reported that Malat1 is highly expressed in iPS (induced pluripotent stem cells). In normal tissues, MALAT1 expresses in a tissue specific patterns, for example, Malat1 is the most abundantly expressed lincRNA in differentiating neurons, and is up regulated in cerebellum, hippocampus and brain stem of human alcoholics [17]. When treated the mouse brain cells with PCP (psychotomimetic agent phencyclidine, which generally induces the perinatal apoptosis and behavioral deficits), Malat1 is highly up regulated [18].

Full length Malat1 localizes in nuclear speckles, which is a dynamic structure that is essential for gathering and recruiting splicing factors [19], thus, Malat1 RNA was considered to be as an important regulatory molecule for trans splicing of RNA [20], Lin M.Y., et al. [16] has found that there is a substantial difference in splices isoforms of lncRNA including Malat1 generated in induced pluripotent stem cells (iPSCs) and neurons, these isoforms are specific to different growth status, the physiological function is unknown. As a major lncRNA, Malat1 also joins regulation process of transcription splicing. Malat1 interacts with serine/arginine splicing factors (SF), results in modulating the distribution of splicing factors in nuclear speckle, and changing cellular levels of phosphorylated forms of SR proteins. Researchers have already identified that Malat1 RNA has multiple physiological functions in normal cells, such as regulating cell cycle [21], Malat1 modulates the expressions of cell cycle genes, which are required for G1/S regulation and mitotic progression with tissue-specific and developmental stage-specific patterns.

In addition to associate with malignant phenotype for high expression of wild type of Malat1 RNA, the mutated forms of Malat1 are also involved in malignant phenotype; for example, t (11; 19) (q13.1; q13.42) was been found in mesenchymal hamartoma of liver [22], this translocation causes breakpoint in Malat1 gene on chromosome 11, thereafter produces mutated forms of Malat1RNA by fusing Malat1 with MHLB1 gene of chromosome 19. t (6;11) (p21;q12) was reported in renal cell carcinoma [23], this translocation causes Malat1 (Alpha)-TFEB gene fusion, results in over expression of native TFEB protein (determined by immuno-histochemistry), while native TFEB in cells without this translocation is not detectable by this assay.

It has been reported that lncRNAs play critical role in normal differentiation/cell fate decision during hematopoiesis. The first lncRNA with key role in hematopoiesis to be described was EGO [24], an evolutionary conserved gene, which transcribes an antisense RNA of ITPR1 gene that modulates the development of eosinophils, EGO is normally expressed in human CD34^+^ hESCs and becomes up regulated during their differentiation into eosinophil. Knockdown of EGO by siRNAs in cultured CD34^+^ progenitors impairs the expression of those genes including major basic protein and eosinophil-derived neurotoxin, which are critical for eosinophil development. Thus, EGO can contribute to eosinophilopoiesis by enhancing the expression of genes needed for this process. lncRNAs are also implicated in the regulation of myelopoiesis, granulocytosis and monocytosis, Zhang et al. [25] identified HOTAIRM1, a gene transcribes an antisense sequence of HOXA1/2 intergenic region, is highly up regulated during retinoic acid-induced granulocytic differentiation of myeloid progenitors. HOTAIRM1 transcript is a ~500 bp RNA fragment, which coordinates along with the activation of HoxA1 and HoxA4 expression during granulocytic differentiation in NB4 cells (acute promyelocytic leukemia cell line) [26]. The understanding regarding involvement of Malat1 in hematopoiesis still remains poor. In this study we are addressed to explore Malat1 expression regulation patterns and possible physiological functions in hematopoietic differentiation.

## Results

### Malat1 is an evolutionary conserved lncRNA and expressed in a wide range of species and tissues of primates

**Fig. 1:**
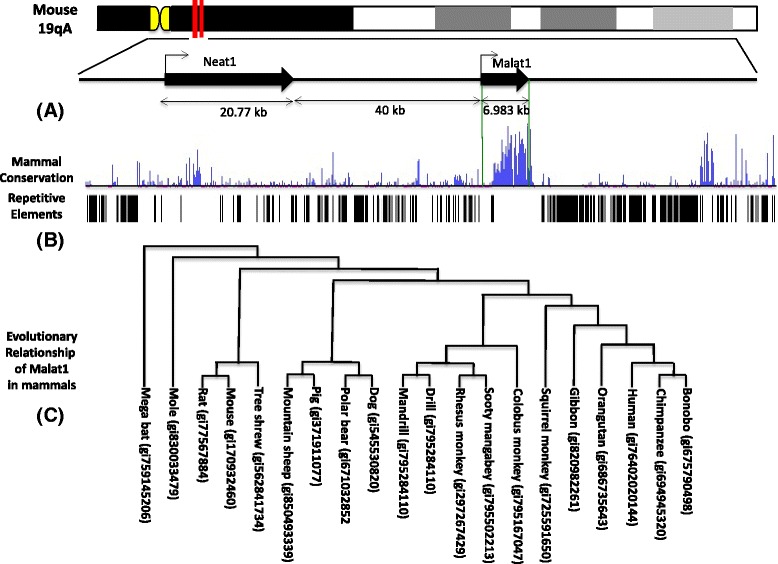
Malat1 gene localization, sequence conservation cross vertebrates and evolutionary Pattern. **a** Mouse Malat1 locus is on chromosome 19 (19A), and is localized on 40 kb down stream of Neat1 gene, yields a 6.983 kb of transcript. **b** Sequence comparison indicates Malat1 gene is the most conserved sequence in around 120 kb region of chromosome 19. **c** Phylogenetic analysis of evolution relationship of Malat1 gene among 20 species of mammals, mouse Malat1 has smallest genetic distance with rat Malat1 and closes to human MALAT1 gene

**Fig. 2:**
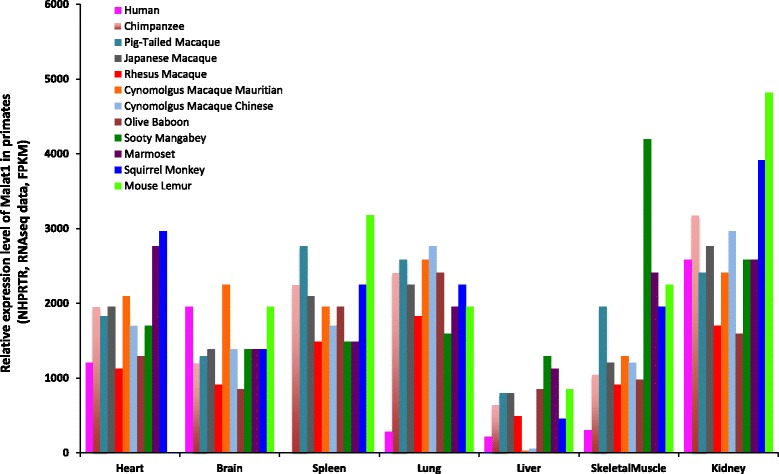
Malat1 expresses in a wide range of species and tissues of primates including human tissue samples. The expression datasets based on RNAseq were used to show the RNA level of Malat1 in different tissues of primates. 7 target tissues were selected as showed in the figure come from 12 different primate species. The Malat1 expression dataset of human tissues are available in NCBI http://www.ncbi.nlm.nih.gov/ieb/research/acembly/av.cgi?db=human, and the expression datasets of other primates are available in the transcriptome database of NHPRTR http://www.nhprtr.org/data/2014_NHP_tissuespecific. The relative expression level of Malat1 RNA was labeled as FPKM (transcript abundances in fragments per kilobase of exon per million fragments mapped)

On mouse chromosome 19, Malat1 gene is located at 40 kb down stream of Neat1 gene (Fig. 1a). Sequence analysis for mouse Malat1 gene shows Malat1 is 6.98 kb transcript, which has been identified both by Northern blot and sequence analysis of transcription. Alignment analysis of multiple genomic sequences shows Malat1 is the most evolutionary conserved sequence in ~120 kb chromosome region (Fig. 1b). The comparison of wide range of species (20 species of mammals) by phylogenetic analysis shows these Malat1 molecules of different resources organisms are from a common ancestor (Fig. 1c). Mouse Malat1 has smallest sequence diversity with rat MALAT1, and human Malat1 is very closed to bonobo and chimpanzee Malat1. Highly conserved coding DNA sequence is common known with important physiological functions, but the role for many of these highly conserved long non-coding DNA sequences remains unclear. The conservative of Malat1 sequence indicates it is a house keeping like gene, more and more evidence shows it up regulates in many cancers, and this research also shows Malat1 is highly expression in undifferentiated hematopoietic stem cells. In addition, several publications have described Malat1 regulates the target gene expression via multiple layer and flexible manners [27]. Therefore, we predict that Malat1 plays a critical regulation role on maintenance of proliferation and malignant cell metastasis ability, more experiments need to be carried out to clarify these functions of Malat1 molecule. Through extensive analysis of publicly accessible panoramic expression databases including GEO (http://www.ncbi.nlm.nih.gov/gds), GeneAtlas (http://biogps.org/dataset), SAGE Genie (http://cgap.nci.nih.gov/SAGE), and NHPRTR (Nonhuman Primate Reference Transcriptome Resource, http://www.nhprtr.org/data/2014_NHP_tissuespecific). We found Malat1 RNA is extensively expressed in different species and tissues of primates including human being. In order to compare the Malat1 expression status in target tissues that we were worked on, we showed the expression abundances of Malat1 RNA in 7 target tissues from 12 primate species based on the datasets of Illumina BodyMap 2 for expression analysis of human Malat1 and NHPRTR for expression analysis of other primates Malat1 (Fig. 2). The Malat1 expresses with high level in other vertebrates such as Zebra fish has been reported recently [28].

### ATRA induces Malat1 up regulation in EML cells

**Fig. 3:**
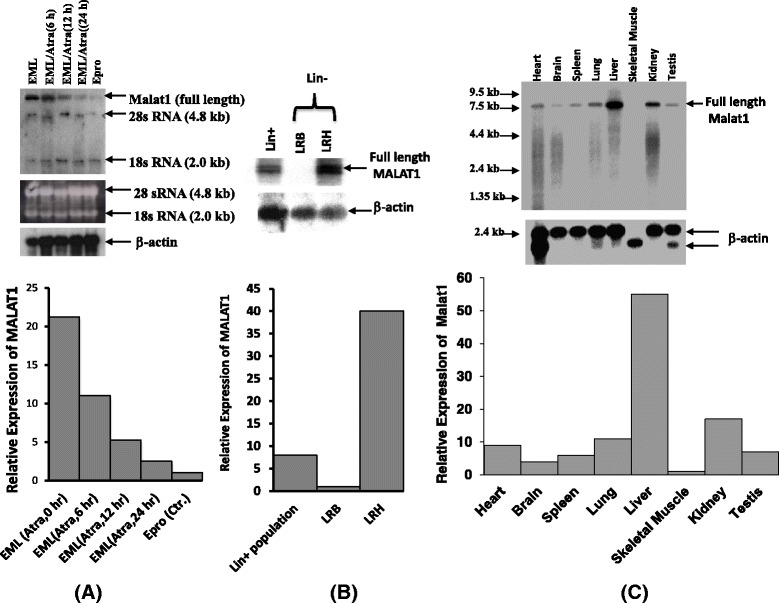
Expression analysis of Malat1 gene in hematopoietic cells. **a** Northern blot showed the expression level of full length Malat1 in ATRA induced EML cells and Epro cells, upper panel: total RNAs from different induction stages of EML cells as designated in the figure. Lower panel: Semi-quantitative analysis of relative expression level for Malat1 gene in indicated EML and Epro cells; **b** Northern blot showed the expression level of full length Malat1 in different populations of mouse bone marrow as indicated in the figure. Upper panel: total RNA from LRH+, LRH-/(LRH) and LRH-/(LRB) were detected with Malat1probe. Lower panel: semi-quantitative analysis of relative expression level for Malat1 gene in these populations. **c** Northern blot showed the expression level for Malat1 gene in multiple tissues of mouse. Upper panel: total RNA from mouse tissues as showed in the figure was detected. Lower panel: semi-quantitative analysis of relative expression level for Malat1 gene in mouse tissues. 225 bp 5’ Malat1 DNA was used as the probe to detect the Malat1 expression, a ~6.9 kb Malat1 RNA was labeled in the figures, the data was been normalized using β-actin, for further details see material and methods

In order to induce EML cell differentiation, we treated the cells with 10 mM of ATRA for 0, 6, 12 and 24 h respectively, total RNAs were detected by Northern blot together with RNA from Epro cells. These RNAs represent the different stages of hematopoietic differentiation, as showed in Fig. 3a (upper panel), a RNA fragment with the size of ~7 kb was highly expressed in EML (0 h) and EML (6 h) and dramatically decreases in EML (12 h) and EML (24 h), tiny expression of this RNA was detected in Epro cells. The quantitative differences of Malat1 expression level among these induction stages were determined by comparison of signal intensity after normalizing to β-actin control. From these comparisons, we conclude Malat1 expression level in EML cells (0 h) is 21.25 times higher than that in Epro; and 8.5, 4 and 2 times higher than that in EML cells exposed to ATRA for 24, 12 and 6 h respectively (Fig. 3a upper and bottom panel).

### Malat1 expresses at high level in early stage of primitive progenitor cells in bone marrow and higher level in liver than in most of other tissues

Malat1 down regulates during hematopoietic differentiation induced by ATRA, if this expression behavior also existed in normal hematopoietic differentiation? We isolated linage negative populations (Lin-) of mouse bone marrow by using the Lin + antibodies cocktails, the Lin-populations was further isolated by using fluorescence dye Rhodamine-123 and Hoechst-33342 as described in material and methods. Our northern analysis shows Malat1 expresses with much higher level in more primitive progenitor cells (LRH), the expression level in LRH population is 40 fold higher than in LRB and 5 fold higher than in Lin + population (Fig. 3b upper and bottom panel). Analysis of multiple tissues shows Malat1 expresses with the highest level in liver, and no detectable expression in skeletal muscle, among tissues assayed in this experiment, the expression level of Malat1 from high to low lists as following: liver > Kidney > lung > heart > testis > spleen > brain > skeletal muscle (See Fig. 3c upper and bottom panel).

### Hematopoietic differentiation induced by ATRA causes up regulation of p53 and inhibition of EML cell growth

**Fig. 4:**
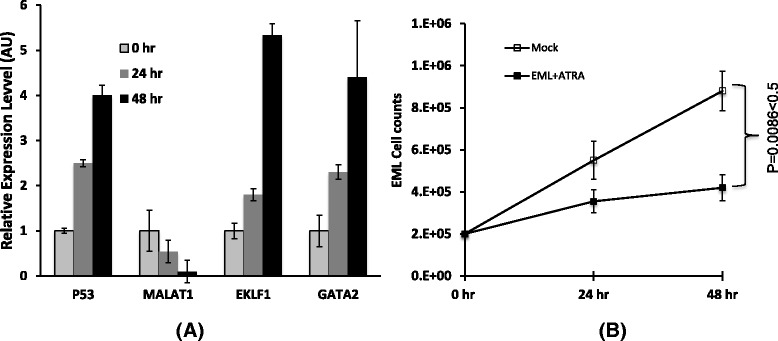
The analysis of gene expression and cell proliferation. **a** EML cell were induced with ATRA for indicated time points. The expression of p53, Malat1, Gata2 and EKLF1 were detected using qRT-PCR, β-actin was used as a loading control, and the means ± SD of three independent experiments are shown. Significant changes were detected from the group of 48 h induction (*P* < 0.05 vs uninduced control). **b** EML cells were treated with or without (mock) ATRA for indicated time points, the graph showed the rate of growth plotted on a liner scale, the means ± SD of three independent experiments are shown, *P* < 0.05 vs Mock control

ATRA treated EML cells induces hematopoietic differentiation [29]. We tested the hematopoietic factor GATA2, which is a critical transcription factor for primitive hematopoiesis procedure [30]. Our data shows that ATRA exposed significantly up regulates GATA2 expression in EML cells (after 48 h exposed, the expression level of GATA2 is up regulated to around 5 times of uninduced EML cells) (Fig. 4a). Since it has been identified that GATA2 is one of the indicator of hematopoiesis of yolk sac stem cells and hematopoietic progenitor cells, therefore ATRA exposed initiates hematopoiesis of EML cells. We were also further tested the expression behavior of EKLF1 (erythroid kruppel like factor), which drives red blood cell differentiation and represses megakaryocyte formation [31, 32]. The results show EKLF1 is up regulated to around 4.5 times of uninduced EML cells (Fig. 4a), that implicates the direction of differentiation is more towards blood cells than megakaryocytes. Analysis for growth rate of ATRA-induced EML cells shows an inhibition effect as showed in Fig. 4b, the proliferation rate of EML cells is decreased around 50 % after 48-h treatment with ATRA (*P* = 0.0086 < 0.05).

### Over expression of p53 down regulates Malat1 expression level via transcriptional inhibition

**Fig. 5:**
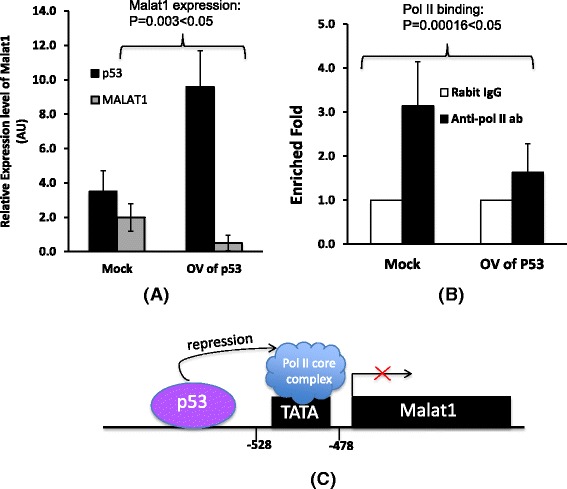
p53 bound to Malat1 promoter and inhibited its expression. **a** Over expression of p53 protein (around 2.7 fold up regulation) in EML cells down regulated Malat1 level with around 4 times, β-actin was used as a loading control, the means ± SD of three independent experiments are shown, *P* < 0.05 (over expression of P53 vs Mock control). **b** CHIP assay to detect the cells with over expression of p53 gene. Rabbit IgG was used as the negative control of CHIP, the means ± SD of three independent experiments are shown, *P* < 0.05 vs Mock control (co-immunoprecipitated with rabbit IgG). **c** Schematic drawing shows RNA polymerase II initiation complex binds to Malat1 promoter region, and p53 protein inhibits DNA binding activity of pol II therefore interferes the initiation complex assembly on transcription start site as indicated in the figure

**Fig. 6:**
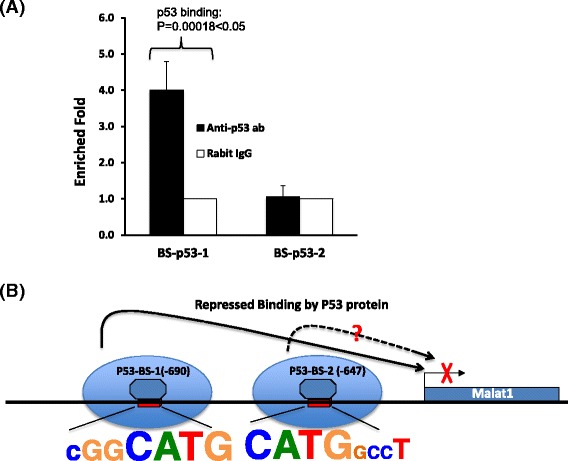
p53 regulated the expression of Malat1 via sequence specific binding. **a** CHIP assay showed p53 protein bound to BS-p53-1 but BS-p53-2 site, the means ± SD of three independent experiments are shown, *P* < 0.05 vs Mock control (co-immunoprecipitated with rabbit IgG). **b** Schematic drawing shows the sequence specific binding of repression. The BS-p53-1 site with conserved sequence of cGGCATG has strong p53 binding activity therefore inhibits Malat1 expression

In order to test if the p53 level in cells affects Malat1 expression, we carried out a transiently transfection experiment that enforced expression of p53 gene in EML cells for 48 h, and then prepared total RNA for qRT-PCR detection. Our data shows p53 expression significantly decreases Malat1 expression level. In this test, when p53 increased to 2.7 times of control, Malat1 decreased around 4 times (*P* = 0.003 < 0.05, Fig. 5a). Based on this observation, we further test if p53 can affect the transcription activity by performing CHIP experiment using above transfected EML cells, the result shows there was less target sequence can be enriched on Malat1 promoter region by anti-pol II antibody (2 fold difference, *P* = 0.00016 < 0.05) in p53 over expressed EML cells (Figs. 5b and 5c). We then predicted the p53 binding sites on promoter region of Malat1 using “PROMO” program (Promoter 2.0 Prediction Server), two typical p53 binding domains have been predicated that the region of (−698 to−669 bp) named as p53-BS-1 locus, the region of (−656 to−625 bp) named as p53-BS-2 locus (Figs. 6a and 6b). We picked up these two sites for CHIP assay experiments by using anti-p53 antibody. Around 4 fold of target sequence was enriched on p53-BS-1 locus (*P* = 0.00018 < 0.05), but no significant enrichment on BS-p53-2 locus.

## Discussion

### Malat1 may preserve the proliferation ability of hematopoietic stem cells

**Fig. 7:**
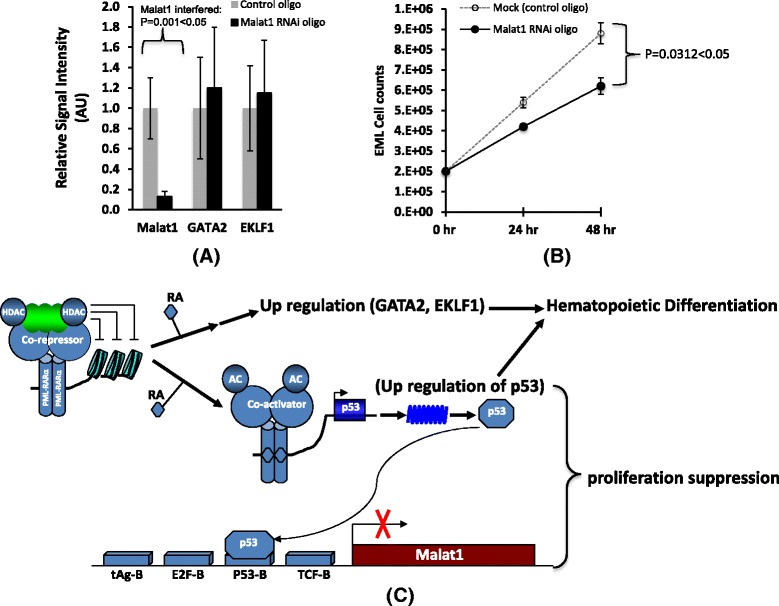
Interfered Malat1 expression didn’t significantly change the hematopoietic differentiation but inhibited the proliferation of EML cells. **a** Inhibited Malat1 expression of EML cells by RNA interference, the decrease of Malat1 didn’t significantly change the expression of hematopoietic factor GATA2 and EKLF1, the means ± SD of three independent experiments are shown, *P* < 0.05 vs Mock control (control RNAi oligo). **b** EML cells were interfered with control RNAi oligo or Malat1 RNAi oligo, cell number was counted at 24 and 48 h, the graph showed the growth rate of EML cells plotted on a liner scale, the means ± SD of three independent experiments are shown, *P* < 0.05 vs Mock control (control RNAi oligo). **c** Schematic drawing shows a work model that ATRA induces hematopoietic differentiation and Malat1 down regulation. In this model, ATRA induces up regulation of GATA2, EKLF1 and tumor repressor p53, and therefore causes hematopoietic differentiation, on the other hand, p53 acts as a transcription factor inhibits Malat1 expression in EML cells, Malat1 played a role in maintaining proliferation potential of hematopoietic cells

As a typical tumor marker, Malat1 expresses with high level in malignant cells. In this study we found Malat1 gene expresses with high level in undifferentiated hematopoietic stem cells and primitive progenitor cells, down regulation of Malat1 accompanies with growth inhibition of EML cells. Our experiments have proved that p53 is acted as a transcription repressor of Malat1 expression to inhibit Malat1 activation. It has been identified that interferes p53 expression inhibits hematopoietic and muscle differentiation [33], reverse function of Malat1 vs. p53 implies Malat1 preserves the ability to enhance the proliferation and inhibit differentiation of hematopoietic and muscle cells. Increased p53 expression causes cell differentiation has been recently suggested by some observations [34–36]. In addition, interferes with endogenous wt-p53 protein of non transformed murine cells 32D (myeloid progenitors) and C2C12 (myoblasts) using dominant negative p53 protein also shows a dramatic inhibition effect of terminal differentiation into granulocytes or myotubes respectively [37]. In this study, we identified that the proliferation ability of hematopoietic cells is significantly inhibited by interfering Malat1 expression in EML cells (Fig. 7b). Taken together, interfering endogenous Malat1 expression directly inhibits cell differentiation, and that Malat1 acts in opposite direction with p53 in EML cells preserves the proliferation ability of hematopoietic stem cells/progenitors.

### p53 protein down regulates Malat1 expression via sequence specific binding

ATRA induces hematopoietic differentiation of EML cells is determined by up regulation of GATA2 and EKLF1 level. Our study has proved this differentiation accompanies with up regulation of p53 and down regulation of Malat1. Surprisingly, p53 protein binds to Malat1 core promoter region, and CHIP data shows p53 can be enriched in the region of (−652 to −765 bp), the core binding loci is in the range of−669 to−698 bp, in which has the sequence of cggCATGgccgccaaggtcgccGTGCCct. Another predicted loci is in the range of (−558 to −670 bp), the core binding loci is in the range of (−625 to −656 bp), in which has the sequence of CATGgccttgctgGGCTGAgaccgcagcct, however, CHIP result shows the second site doesn’t effectively bind with p53 (Fig. 5b). Therefore we speculate even both sites (p53-BS1 and p53-BS2) have conserved CATG sequence domain, but only p53-BS1 binds to p53 protein and inhibited Malat1 gene activation, hence the background sequence of p53-BS1 with conserved domain CATG may favor a configuration of p53 binding. Our finding is consistent with some expression study of p53, for example, viral protein dysregulated p53 expression leads to up regulation of Malat1, and mutation or inactivation of p53 is also shown up regulation of Malat1 expression [38]. Therefore we conclude that p53 is a negative regulator of Malat1, over expression of p53 may play a therapy effect to target inhibition of oncogenic Malat1 level in cells.

### Proliferation suppression by down regulation of Malat1 in hematopoietic cells

Apparently, our study has proved Malat1 down regulates in late stage hematopoietic progenitors and more differentiated primitive cells, and Malat1 gene expression is inhibited by tumor repressor p53 protein. If down regulation of Malat1 leads to hematopoietic differentiation via proliferation inhibition? In order to answer this question, we interfered Malat1 expression of EML cells, in this study Malat1 was down regulated with 7–8 fold (*P* = 0.001 < 0.05), but hematopoietic differentiation marker GATA2 and EKLF1 didn’t significantly change the expression level (*P* = 0.15 > 0.05 for GATA2 comparison and *P* = 0.13 > 0.05 for EKLF1 comparison. Fig. 7a). Hence over expression of Malat1 can’t directly drive the hematopoietic differentiation, however, the down regulation of this gene can significantly reduced hematopoietic cell (EML) proliferation rate (*P* = 0.0312 < 0.05, Fig. 7b), whereas, we speculate one of the ability for Malat1 is maintained the proliferation potential of hematopoietic stem cells/progenitors. This result is also consistent with the studies from knockdown of Malat1 in myoblast [39, 40]. Based on these studies we proposed a model regarding Malat1 function (Fig. 7c), during hematopoietic differentiation of EML cells by retinoic acid exposure, RA induces up regulation of GATA2 and EKLF1, then initiates hematopoietic differentiation; in the meantime, up regulation of p53 also results in regulation of downstream gene expression including negative regulation of Malat1, thus causes proliferation suppression. The mechanisms for Malat1 RNA regulating cell proliferation are still unclear.

## Conclusions

Malat1 is one of the most significant molecules of long non-coding RNA (lncRNA), which is commonly up regulated in malignant cells, hence it is usually considered as a biomarker of some type of cancers. Our research has clarified the aspects of normal biological function and transcriptional regulation during hematopoiesis. A, Malat1 down regulates during hematopoietic differentiation induced via ATRA; B, p53 is a negative regulator of Malat1 expression through binding to its promoter region; C, Malat1plays a critical role in maintaining the proliferation potential of early-stage hematopoietic cells. In summary, our research is the first report that Malat1 plays a role in maintaining the proliferation potential of hematopoietic cells. And Malat1 is transcriptionally regulated by tumor suppressor p53 during ATRA induced hematopoiesis.

## Methods

### Cell culture

EML cells (Multipotent hematopoietic cell line, progenitor cells of myeloid, erthroid and megakaryocytic and B-lymphoid lineages) were cultured with Iscove’s Modified Dulbecco’s Media (IMDM), supplemented with 20 % heat inactivated horse serum, and conditioned medium from cultured BHK/MKL cells was used as the source of stem cell factors. EML cells were induced to differentiate by 10 μM all-trans retinoic acid (ATRA) for indicated time points as showed in figures (Fig. 2a upper and bottom). Epro cells were cultured with IMDM containing 20 % horse serum and 10 % HM-5 conditioned medium as the source of mouse GM-CSF. All growth mediums contained 5 U/ml penicillins, 5 μg/ml streptomycin sulfate, and 2 mM l-glutamine (Invitrogen).

### RNA interferences (RNAi)

Malat1 expression was silenced by RNA interference, briefly, used the BLOCK-iT™ online program to pick up DNA sequences that locate in ORF region of mouse Malat1 gene as following: MA-siRNA-285: GCAGTTTAGGAGATTGTAA (control oligo: MA-ctr-siRNA-285: GCAGATTAGAGGTTTGTAA); MA-siRNA-2247: GCGGAATTGCTGGTAGTTT (Control oligo: MA-ctr-siRNA-2247: GCGGTTAGGTCGATAGTTT). Synthesized double strand RNAs for these oligos and transiently transfected into EML cells as following: 30 μl of Lipofectamin 2000 was mixed with 1.5 ml of Opiti-MEM medium, then incubated at RT for 5 min, mixed 75 μl of double strand RNA (20 μM) with 1.5 ml of Opiti-MEM medium, incubated at RT for another 5 min. Mixed RNAs and Lipofectamine solution and incubated at RT for total 20 min. Harvested EML cells with spin and then resuspended **~**2X107cells with 1.5 ml of Opiti-MEM medium, added RNA-Lipofectamin mixture to these cells and mixed gently, then incubated for 48 h in a CO_2_ incubator.

### Chromatin precipitation (CHIP) and promoter binding assay

ChIP assays were performed using chromatin immunoprecipitation assay kit (Upstate) following manufacturer's instruction. EML cells (2X106) were plated per 100-mm dish before 24 h transfection, and then transfected with 24 μg indicated plasmids. After 48 h transfection, harvested cells and cross linked with fixing buffer (1 % formaldehyde, 10 mM NaCl, 50 mMHePes, pH7.5, 1 mM EDTA, 0.5 mM EGTA), prepared nuclear extracts and then fragmentized chromatin DNA into an average of 200 bp segments by sonication. Chip assays were performed with anti-p53 and anti-pol II antibodies, reversed precipitated DNA-protein complex and purified DNA fragments, then performed quantitative PCR using QuantiTect SYBR Green PCR master mix (Qiagen). Primers were used as following: for p53 binding site assay: Site 1: FMA53-1, gatagacctggggaccttgc, RMA53-1, atggtcgctgcaggtgag, the amplified fragment was 113 bp; Site 2: FMA53-2, ctcacctgcagcgaccat, RMA53-2: ctctcggggccacttttc, and the amplified fragment was 112 bp. For pol II binding site assay, Fpol: tcgtccctacaggagcattc, Rpol: cagcttcagcttccacttcc, and the amplified fragment was 160 bp.

### RNA isolation, Northern blot and qPCR /qRT-PCR

Total RNA was isolated from EML, EPRO and isolated bone marrow cells using trizol reagent (Life Technologies, Gaithersberg, MD). Northern blot was performed with 10 μg total RNA per lane. Probe for detecting Malat1 was 225 bp of 5’ sequence (spanning 72-296 bp of the sequence of Genebank Accession number HG982261.1). Probe for detecting β-actin was 202 bp of 5’ sequence (spanning 490–691 bp of the sequence of Genebank Accession number X03672). A mouse multiple tissue RNA blot was purchased from Clontech. The probes were labeled using PCR amplification that ^32^P-labeled dCTP was incorporated into produced PCR fragments. RNA was electrophoresed on a 1 % agrose/formaldehyde gel and was blotted onto membranes (Amersham Pharmacia Biotech) followed by UV crosslinking. The hybridization was performed at 65 °C for 16 h. Signals on the washed membrane were visualized by autoradiography and were quantitated by densitometry analysis. The qPCR or qRT-PCR was performed as previously described.

### Purification of LRH and LRB cells from mouse bone marrow

Prepared bone marrow cells from 8–12-week-old BALB/c mice, then depleted lineage positive cells by using a mixture of phycoerythrin (PE)**–**conjugated monoclonal antibodies directed against surface antigens (derived from blood cells including CD11b, CD5, CD8a, CD4, Gr-1, CD45R, Ter119) were purchased from BD Biosciences (San Jose, CA) and PE magnetic beads were purchased from Miltenyi Biotech (Surrey, U.K.). Purified Lin (-) cells were then further purified using a cocktail solution of supravital dyes. Principally, Lin- cells were suspended in FACS stain buffer (1XHBSS buffer) containing Rhodamine-123 and Hoechst-33342 for 15 min; labeled cells were then sorted on FACS-STAR (Becton Dickinson), the Lin-Rho^low^/Hoechst^Bright^(LRB) represented late stage progenitor cells and the Lin-Rho^low^/Hoechst^Low^(LRH) represented more primitive progenitor cells, were highly enriched from Lin- population and used for Malat1 expression analysis.

### Statistical analysis

The mean value and standard deviation of three independent experiments were used. P values were calculated with a paired student’s *T* test (two tailed hypothesis). A *P* value of < 0.05 between experimental sample (group) and control sample (group) was considered to be statistically significance.

### Availability of supporting data

The Malat1 sequences and the phylogenetic tree of 20 species of mammals in Fig. 1c are available in Dryad repository, DOI: doi:10.5061/dryad.017t8.

The expression datasets of 7 tissues from 12 different primate species in Fig. 2 are from http://www.nhprtr.org/data/2014_NHP_tissuespecific and http://www.ncbi.nlm.nih.gov/ieb/research/acembly/av.cgi?db=human.

## Abbreviations

ATRA: All-trans Retinoic Acid
ChIP: Immunoprecipitation
EML: Erythroid myeloid lymphoid
hESC: Human embryonic stem cell
iPSC: Induced pluripotent stem cell
lncRNA: Long non-coding RNA
SR proteins: Serine/arginine (SR)-rich Proteins
